# Novel Differentially Expressed LncRNAs Regulate Artemisinin Biosynthesis in *Artemisia annua*

**DOI:** 10.3390/life14111462

**Published:** 2024-11-12

**Authors:** Tingyu Ma, Tianyuan Zhang, Jingyuan Song, Xiaofeng Shen, Li Xiang, Yuhua Shi

**Affiliations:** 1 Key Lab of Chinese Medicine Resources Conservation, State Administration of Traditional Chinese Medicine of the People’s Republic of China, Institute of Medicinal Plant Development, Chinese Academy of Medical Sciences & Peking Union Medical College, Beijing 100193, China; tyma@implad.ac.cn (T.M.); jysong@implad.ac.cn (J.S.); xfshen@implad.ac.cn (X.S.); 2 Genome Analysis Laboratory of the Ministry of Agriculture and Rural Affairs, Agricultural Genomics Institute at Shenzhen, Chinese Academy of Agricultural Sciences, Shenzhen 518120, China; zhangtianyuan@caas.cn; 3 Key Laboratory of Beijing for Identification and Safety Evaluation of Chinese Medicine, Institute of Chinese Materia Medica, China Academy of Chinese Medical Sciences, Beijing 100700, China

**Keywords:** *Artemisia annua*, artemisinin biosynthesis, long non-coding RNA

## Abstract

Long non-coding RNAs (lncRNAs) are crucial in regulating secondary metabolite production in plants, but their role in artemisinin (ART) biosynthesis, a key anti-malarial compound from Artemisia annua, remains unclear. Here, by investigating high-artemisinin-producing (HAP) and lowartemisinin-producing (LAP) genotypes, we found that the final artemisinin content in *A. annua* is influenced by the quantity of the precursor compounds. We report on RNA deep sequencing in HAP and LAP genotypes. Based on the application of a stringent pipeline, 1419 novel lncRNAs were identified. Moreover, we identified 256 differentially expressed lncRNAs between HAP and LAP. We then established correlations between lncRNAs and artemisinin biosynthesis genes in order to identify a molecular framework for the differential expression of the pathway between the two genotypes. Three potential lncRNAs (MSTRG.33718.2, MSTRG.30396.1 and MSTRG.2697.4) linked to the key artemisinin biosynthetic genes (*ADS*: Amorpha-4,11-diene synthase, *DXS*: 1-deoxy-D-xylulose-5-phosphate synthase, and *HMGS*: 3-hydroxyl-3-methyglutaryl CoA synthase) were detected. Importantly, we observed that up-regulation of these lncRNAs positively modulates the target artemisinin biosynthetic genes, potentially leading to high artemisinin biosynthesis in HAP. In contrast, *BAS* (beta-amyrin synthase), which is involved in the artemisinin competing pathway, was strongly down-regulated in HAP compared to LAP, in line with the expression pattern of the linked lncRNA MSTRG.30396.1. By identifying and characterizing lncRNAs that are potentially linked to the regulation of key biosynthetic genes, this work provides new insights into the complex regulatory networks governing artemisinin production in *A. annua*. Such findings could pave the way for innovative approaches in metabolic engineering, potentially enhancing artemisinin yields and addressing challenges in sustainable production.

## 1. Introduction

The main natural source of Artemisinin, a sesquiterpene lactone with a peroxide bridge, is the medicinal herb *Artemisia annua* [1]. Artemisinin is produced in glandular secretory trichomes (GSTs), which are specialized 10-cell structures located on the surface of the leaves, stems and flower buds of *A. annua* [2]. It is the primary component of the WHO-recommended malaria treatment. The biosynthetic pathway of artemisinin has been the subject of extensive research, driven by scientific interest and its significant potential for meeting pharmaceutical market demand [3]. Several regulatory enzymes involved in the biosynthetic pathway have been found and tested to augment the artemisinin content [4]. These enzymes include, among others, amorpha-4,11-diene synthase (*ADS*), farnesyl diphosphate synthase (*FPS*), *CYP71AV1*, *DBR2*, cytochrome P450 reductase (*CPR*) and aldehyde dehydrogenase 1 (ALDH1) [5]. However, artemisinin is naturally synthesized in limited quantities, resulting in a global supply shortage [6].

Chemical synthesis of artemisinin is challenging due to its complex configuration. As a result, *A. annua* remains the primary commercial source of artemisinin [7]. Recent advances in genetic and metabolic manipulation have led to a plethora of different strategies and innovations aimed at enhancing the in vivo synthesis of artemisinin, not only in *A. annua* but also in alternative plant species [5]. Co-expression of FPS, CYP71AV1 and its electron transfer partner POR resulted in a 3.6-fold increase in artemisinin production [8]. Meanwhile, co-expression of ADS, CYP71AV1, ALDH1 and POR resulted in a 3.4-fold increase in artemisinin levels [9]. Additionally, the up-regulation of *DBR2* clearly demonstrated its pivotal role as a critical enzyme in controlling artemisinin production by directing the metabolic flux from artemisinic acid to dihydroartemisinic acid [10]. In the absence of DBR2 activity, *A. annua* exclusively synthesizes artemisinic acid, resulting in the exclusive formation of arteannuin B [10], highlighting the importance of *DBR2* in modulating the artemisinin biosynthetic pathway.

Long non-coding RNAs (lncRNAs) have been recognized as important regulators of gene expression in various biological processes, including the biosynthesis of plant metabolites [11]. While much research has primarily focused on protein-coding genes involved in the biosynthesis of plant metabolites, emerging evidence suggests that lncRNAs also play significant roles in regulating the biosynthesis of plant specialized metabolites including flavonoids, terpenoids and alkaloids [12]. In *Ginkgo biloba*, lncRNAs have been reported to target both structural (*PAL*, *ANR*, *F3H*, *CHI*) and transcription factor genes (*WD40*, *bHLH*, *MYB*) involved in flavonoid biosynthesis [13,14]. In *Cinnamomum camphora*, a set of 17 differentially expressed lncRNAs were found to be linked to sesquiterpene and monoterpene biosynthesis [15]. In *Nicotina tabacum*, the lncRNA *NTA-ETMX27* was reported to act as an endogenous mimic by slowing down the target of the *QPT2* gene, which affects nicotine accumulation [16,17,18]. Bordoloi et al. [19] reported the expression of six lncRNAs associated with genes involved in the metabolism of terpenoids and flavonoids in *Citrus limon*.

This aim of this study is to systematically investigate how lncRNAs might influence gene expression related to artemisinin biosynthesis in *A. annua*. While previous research has predominantly focused on the characterization of enzymatic pathways and transcriptional factors involved in artemisinin synthesis, the role of lncRNAs remains largely unexplored. To achieve this objective, we took advantage of HPLC-MS-based metabolic profiling coupled with RNA sequencing to (i) measure the artemisinin content in two *A. annua* contrasting genotypes (a high-artemisinin-producing genotype termed HAP, and a low-artemisinin-producing genotype named LAP), (ii) identify lncRNAs in the transcriptomes of the two genotypes, (iii) assess their differential expression, (iv) investigate their functional properties and target genes and (v) test the expression level of potential candidate lncRNAs by semi-quantitative PCR. This research not only advances our understanding of non-coding RNA biology in medicinal plants but also opens up new avenues for improving the biosynthesis of pharmacologically valuable compounds.

## 2. Materials and Methods

### 2.1. Plant Material Preparation

Two *A. annua* genotypes were utilized in the current study: a high-artemisinin-producing type, referred to as HAP, and a low-artemisinin-producing type, termed LAP. The seeds of HAP and LAP were collected from Sanjiazi Township, Yilaxi Town, Yongji County, Jilin Province (E126°0′26.22″, N43°47′15.14″), and Hainan Qiongzhong Jiacha farm (E109°46′23.16″, N19°02′29.00″), in 2017 and 2016, respectively. Subsequently, the collected seeds were sown in a greenhouse (with the temperature maintained at 25 ± 2 °C) in the field located at Huairou, Beijing, in March and harvested at the end of July 2018. Three individual plants of each type, exhibiting uniform morphology, were carefully selected, and the leaves from each main stem were collected and rapidly frozen in liquid nitrogen. All collected materials were partially stored at −80 °C, with the remainder being subjected to lyophilization.

### 2.2. Metabolite Analysis by Ultra-High-Performance Liquid Chromatography–Mass Spectrometry

By employing an HPLC-MS method, the concentrations of artemisinic aldehyde, artemisitene, dihydroartemisinic acid, artemisinin acid, artemisinin B and artemisinin in the samples were determined. In brief, the lyophilized tissues of JAP and HAP were ground into powder. For each 50 mg sample powder, 2.5 mL of methanol was added, and the extraction was performed using an ultrasonic (KQ-500DE) method (at 40 kHz) for 15 min (Kunshan Ultrasonic, Suzhou, Jiangsu, China). The resulting supernatant was utilized to determine artemisinic aldehyde and artemisitene concentrations. However, for the analysis of dihydroartemisinic acid, artemisinin acid, artemisinin B and artemisinin, the supernatant was diluted 100 times before use. All extracts were filtered through a 0.22 μm dimension micropore film and subsequently analyzed using a 1290-6470 UPLC-APCI MS/MS system from Agilent (Santa Clara, CA, USA).

The UPLC analytical conditions were set as follows: the HPLC column used was Eclipse Plus C18, RRHD 2.1 × 50 mm, with a particle size of 1.8 µm (Agilent Technologies, Santa Clara, CA, USA). The solvent system consisted of phase A, which was ultrapure water containing 0.1% formic acid and 5 mM ammonium formate, and phase B, which was methanol. The gradient program initiated with 55% of phase B at 0 min increased to 100% at 8 min, was maintained at 100% until 11 min and then reverted to 55% at 14 min. The flow rate was set to 0.6 mL/min, and the temperature was maintained at 40 °C (Figure S1). The injection volume used was 5 µL [20].

### 2.3. RNA Extraction, Library Preparation, Sequencing and Transcriptome Assembly

Total RNA was extracted from 5-month-old plant leaf with Trizol reagent (Invitrogen, Carlsbad, CA, USA). mRNA was purified using the Oligotex mRNA Midi Kit (Qiagen, Hilden, Germany). The mRNA quantity and quality were assessed with the Invitrogen Qubit 2.0 and Agilent 2100 (Agilent Technologies, Santa Clara, CA, USA). Additionally, 3 μg of RNAse per sample was used to remove ribosomal RNA (rRNA) with the Epicentre Ribo-Zero rRNA Removal Kit (Epicentre, Madison, WI, USA). Following ethanol precipitation to clean the rRNA-free residue, sequencing libraries were constructed using the rRNA-depleted RNA and an NEBNext Ultra Directional RNA Library Prep Kit for Illumina (NEB, Ipswich, MA, USA), according to the manufacturer’s instructions. Library quality was assessed before using a cBot Cluster Generation System to cluster the index-coded samples with a TruSeq PE Cluster Kit v3-cBot-HS (Illumina), following the manufacturer’s recommendations. The libraries were then sequenced on the Illumina Hiseq xten platform, generating 150 bp paired-end reads [21]. For the assessment of differentially expressed lncRNAs, RNA-seq data for each genotype was performed in triplicate. Raw reads were deposited under the NCBI project PRJNA996091.

A sequencing data quality check and trimming were carried out with FastQC v0.12.1 [22] and Trimmomatic v0.39 [23], respectively. Clean reads were then mapped to the genome of *A. annua* cv Huhao1 (GenBank assembly accession: GCA_003112345.1) [24] using STAR v2.7.9a [25]. Subsequently, transcript assembly for each genotype was carried out with Stringtie v2.2.1 [26].

### 2.4. Bioinformatics Pipeline for Identification of LncRNAs

According to the structural characteristics and non-coding functional features of lncRNA, some filter conditions were set to screen lncRNAs. Briefly, we discarded transcripts shorter than 200 bp with a mapping read count > 10. Besides transcripts exhibiting FPKM ≥ 1, transcripts in at least one sample were retained. Transcripts without coding potential were identified using the Coding Potential Calculator (CPC) tool (CPC score < 0) [27]. We double-checked with the Coding-Non-Coding Index (CNCI) tool [28] (CNCI score < 0). Subsequent final lncRNAs were used for downstream analysis.

### 2.5. Differential Expression of LncRNAs

The quantification of lncRNA expression was determined using the Fragments Per Kilobase of transcript per Million mapped reads (FPKM) metric through the HTseq-count 0.6.0 [29]. We considered an expression to be significantly differentially expressed if its q-value was less than 0.05, and the absolute value of Foldchange ≥ 2. DESeq2 R package (v1.12.3) was used to compare DEGs.

### 2.6. Target Gene Prediction

LncRNAs primarily act in a cis- or trans-regulatory manner on target genes, leading to their categorization into two cases for predicting lncRNA target genes. Subsequently, we conducted a search for coding genes located 10 kb/100 kb upstream and downstream of lncRNAs, considering them as the cis target genes. Regarding the trans-acting lncRNAs, the target genes were identified based on the correlation of expression level between lncRNAs and coding genes with custom scripts. If the absolute value of the correlation coefficient exceeded 0.95, we utilized functional enrichment analysis with the KOBAS-i tool (v3.0) [30] to predict the functions of lncRNAs based on their associated target genes.

### 2.7. Real-Time Quantitative PCR

cDNA was synthesized from 1 μg of total RNA using the PrimeScript II 1st Strand cDNA Synthesis Kit (Takara, Dalian, China), following the manufacturer’s instructions. The cDNA samples were then diluted 30-fold for use as templates in quantitative real-time PCR (qRT-PCR) analysis. qRT-PCR was conducted using the cDNA with 2× SYBR^®^ Premix Ex Taq ™ II (Tli RNaseH Plus) (Takara, Dalian, China) on a Roche LightCycler 96 real-time PCR machine (Roche, Germany). β-Actin served as a reference gene to normalize expression and calculate the 2^−ΔΔct^ value [31]. Three independent experiments were performed for each sample.

## 3. Results

### 3.1. Metabolite Profiling of HAP and LAP Genotypes of Artemisia annua

**Figure 1:**
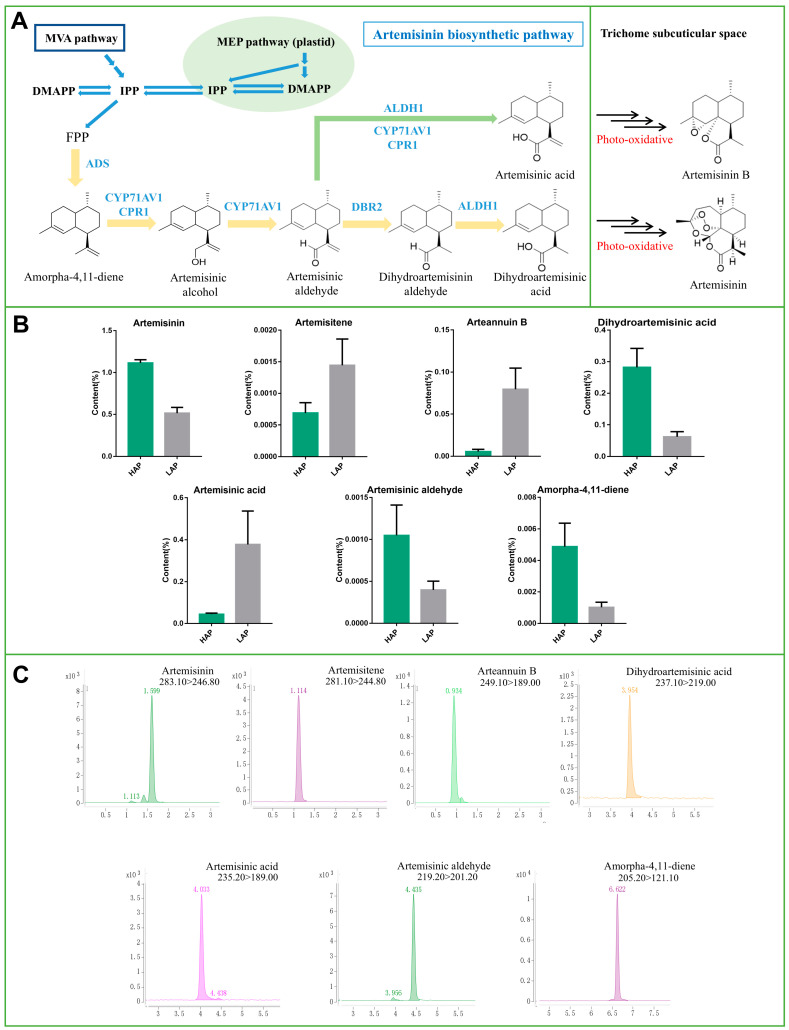
A simplified biosynthesis pathway in *A. annua* showing the route of synthesis of artemisinin and artemisinin B biosynthesis (**A**) Metabolite content in the two *A. annua* genotypes: HAP (high-artemisinin-producing type) in green, and LAP (low-artemisinin-producing type) in gray (**B**). A chromatogram for the seven studied metabolites including artemisinin, artemisitene, arteannuin B, dihydroartemisinic acid, artemisinic acid, artemisinic aldehyde and amorpha-4,11-diene (**C**).

LAP and HAP are different genotypes of *A. annua*. To ensure the accuracy of the change in content in the artemisinin biosynthesis pathway (Figure 1A), seven related compounds were quantified by the UPLC-QQQ(APCI)-MS/MS method. The metabolite analysis revealed that artemisinin was the most abundant sesquiterpenoid detected in *A. annua* leaf extracts (Figure 1B,C). HAP was highly enriched in artemisinin with a concentration reaching up to 1.1% of extracted leaf dry weight (Figure 1B,C). In addition, the content of artemisinin in the final product in HAP was 2.38 times that of LAP. HAP tends to synthesize more dihydroartemisinic acid than artemisinic acid. On the contrary, LAP strongly synthesized artemisinic acid. Therefore, the content of arteannuin B in LAP is 20.4 times that of HAP. Furthermore, Amorpha-4,11-diene and Artemisinic aldehyde, which are the common precursors of artemisinin and arteannuin B, were highly accumulated in HAP, indicating that the final artemisinin content in *A. annua* is influenced by the quantity of the precursor compounds.

### 3.2. Identification of LncRNAs in Artemisia annua Genome

To identify lncRNAs in *A. annua*, we generated RNA sequencing data from low-artemisinin-producing (LAP) and high-artemisinin-producing (HAP) leaf samples, which came from the same harvest period. A total of 489 million clean reads (73.36 Gbp) were generated and the alignment results are shown in Table S1. The Q30 was >93%, indicating that our data were of high quality. The mapped ratio of each sample ranged from 75.14% to 81.79%.

**Figure 2:**
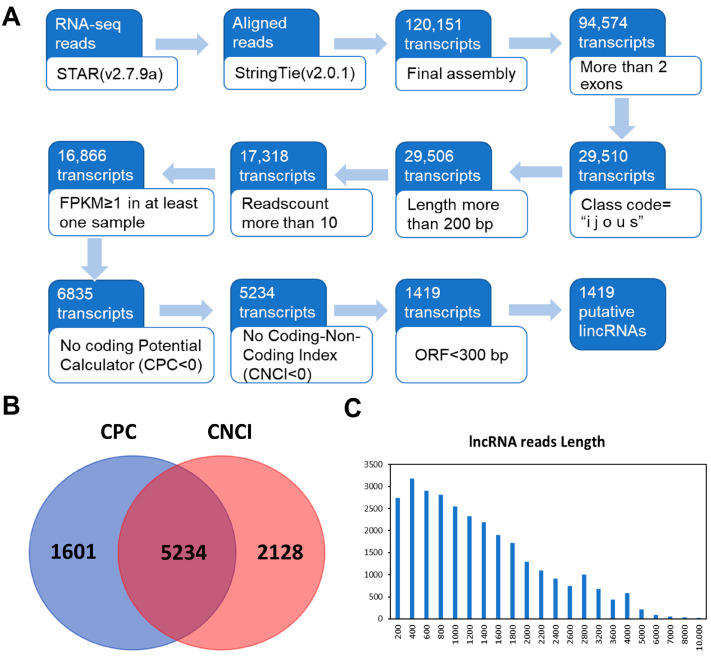
Genome-wide identification of lncRNAs in *Artemisia annua*. Flowchart of prediction of novel lncRNAs in *A. annua* (**A**). Venn diagram showing shared lncRNAs from CPC and CNCI prediction tools (**B**). Distribution of lncRNAs within *A. annua* genome (**C**).

Utilizing the detailed methodology (Figure 2A) outlined in Section 2, we successfully identified the lncRNAs of *A. annua*. We performed short-read gapped alignment using STAR v2.7.9a [25] onto the *A. annua* genome. Therefore, we used the publicly available assembly software StringTie v2.0.1 [26] to reconstruct the transcriptome de novo for each sample based on the read-mapping results. We first compared transcripts with known genes and obtained a set of potential intergenic transcripts. Then, we filtered by the exon number, transcript length, class code and read count number, and acquired 29,510 potential long non-coding transcripts.

Using an FPKM expression level of more than one at least in one sample as a basis, we finally obtained 16,866 reliably putative long non-coding transcripts. After the data were filtered with CPC [27] and CNCI [28], transcripts with an ORF length less than 300 bp were retained from LAP and HAP data (Figure 2B). The CPC [27] and CNCI [28] tools were used to simultaneously assess the coding potential of 6835 transcripts, leading to the identification of 1419 newly discovered transcripts. CPC [27] and CNCI [28] analyses yielded a total of 5234 shared lncRNAs (Figure 2B), evenly distributed across the 20 chromosomes of *A. annua* genome (Figure 2C).

### 3.3. Differentially Expression Profiles of LncRNAs in LAP and HAP

**Figure 3:**
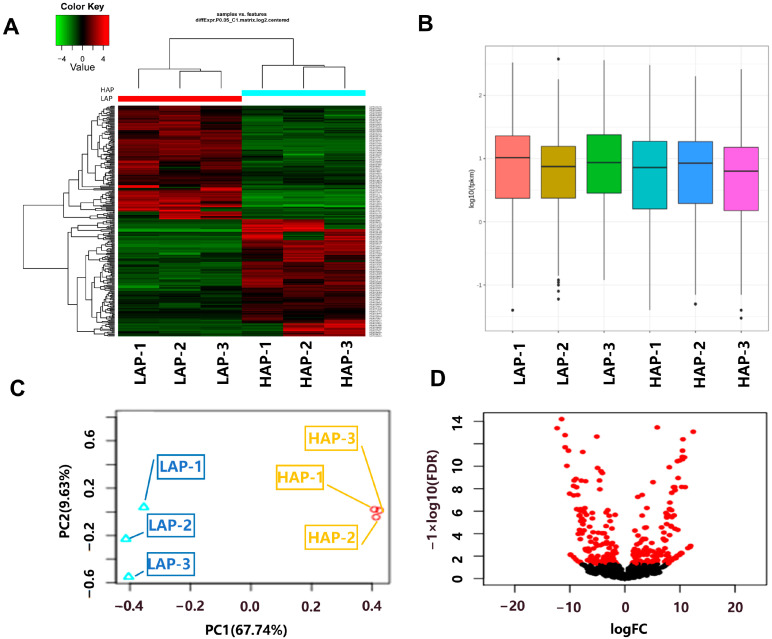
Expression overview of lncRNAs from *Artemisia annua* genotypes HAP (high-artemisinin-producing type) and LAP (low-artemisinin-producing type). Heatmap of expressed lncRNAs (**A**). Boxplot showing log10(FPKM) value of lncRNAs (**B**). Principal component analysis depicting LAP and HAP projection on two first principal component axes (**C**). Volcano plot of exhibiting differentially expressed, up-regulated and down-regulated lncRNAs from HAP vs. LAP analysis. Red dots: lncRNAs with a q-value < 0.05 and a significant differential expression. Black dots: lncRNAs without a significant difference expression (**D**).

Comparative lncRNA expression profiling was conducted between HAP and LAP genotypes (Figure 3). The heatmap (Figure 3A) of the expression data shows a clear distinction of LAP and HAP genotypes. While the log value of the FPKM values followed an approximately similar variation range (Figure 3B), the principal component analysis (Figure 3C) clearly classified HAP and LAP genotypes in two distinct groups, suggesting the reliability of the selected samples.

Overall, 4596 differentially expressed genes (DEGs) were identified while 264 differentially expressed lncRNAs (DELs) were identified when comparing HAP vs. LAP. Among the 264 DELS, 130 DELs were found to be up-regulated and 134 were down-regulated (Figure 3D).

### 3.4. Roles of LncRNAs in Both Cis- and Trans-Regulation of Target Genes

To investigate the role of lncRNAs in biological processes, we aimed to predict their effects on cis and trans target genes. For the cis-acting lncRNAs, we examined protein-coding genes located within 100 kb upstream and downstream of lncRNAs. Our results showed that 240 lncRNAs can potentially regulate 755 protein-coding genes, forming a total of 744 gene pairs (Table S2).

**Figure 4:**
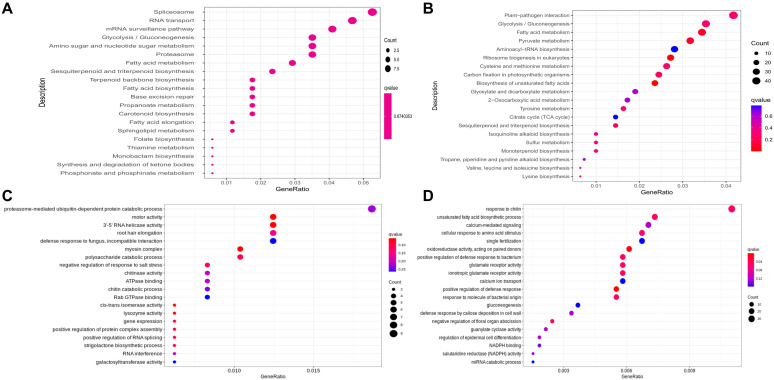
Common and unique enriched KEGG and GO enrichment of target genes of cis- and trans-lncRNAs in HAP (high-artemisinin-producing type) and LAP (low-artemisinin-producing type) genotypes. (**A**) Common enriched KEGG pathways of lncRNAs cis-acting target genes. (**B**) Common enriched KEGG pathways of two genotypes of lncRNA trans-acting target genes. (**C**) Unique enriched GO terms of cis-acting target genes. (**D**) Unique enriched GO terms of lncRNA trans-acting target genes.

The trans-acting lncRNAs and their corresponding protein-coding genes were predicted by evaluating the correlation coefficient of expression using a cut-off of 0.9. As a result, a total of 144 trans-acting lncRNAs were found with a predicted number of 28,276 gene pairs (Pearson correlation ≥ 0.95 or ≤−0.95). Furthermore, functional enrichment analysis of the predicted cis- and trans-acting lncRNAs target genes was carried out to find out the related biological processes (Figure 4).

Functional KEGG enrichment of both cis- and trans-lncRNAs target genes revealed their possible involvement in the regulation of metabolite biosynthesis, such as sesquiterpenoid and triterpenoid (Figure 4A,B). Meanwhile, GO enrichment suggested that the lncRNAs target genes are involved in root hair elongation, the defense response to fungus, the strigolactone biosynthetic process (Figure 4C), the unsaturated fatty acid biosynthetic process, the defense response to bacterium and the miRNA catabolic process (Figure 4D).

**Figure 5:**
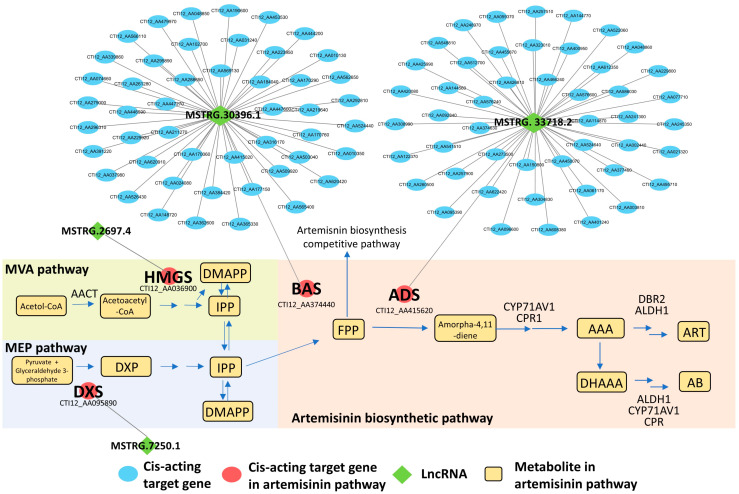
Analysis of cis-acting lncRNAs and their cis-targeted genes. Amorpha-4-1-diene synthase (*ADS*), beta-amyrin synthase (*BAS*), 1-deoxy-D-xylulose-5-phosphate synthase (*DXS*) and 3-hydroxyl-3-methyglutaryl CoA synthase (*HMGS*).

Since artemisinin is a sesquiterpene lactone with a unique endoperoxide structure, in-depth analyses of target genes possibly involved in terpenoids biosynthesis were performed based on functional and correlation analyses. According to Table S2, we found that a total of four cis-acting lncRNAs (MSTRG.33718.2, MSTRG.7250.1, MSTRG.30396.1 and MSTRG.2697.4) had target genes related to artemisinin biosynthesis (Figure 5, Table S3). The Pearson correlation coefficient between MSTRG.33718.2 and the target gene amorpha-4-1-diene synthase (*ADS*) was 0.98. MSTRG.30396.1 was detected to be associated with beta-amyrin synthase (*BAS*) with a Pearson correlation of 0.98. The target gene of MSTRG.7250.1 was predicted to be 1-deoxy-D-xylulose-5-phosphate synthase (*DXS*) with a Pearson correlation > 0.95. Similarly, MSTRG.2697.4 was linked to 3-hydroxyl-3-methyglutaryl CoA synthase (*HMGS*) with a Pearson correlation > 0.95 (Figure 5).

### 3.5. Quantitative RT-PCR Validation of Selected LncRNAs and Genes

**Figure 6:**
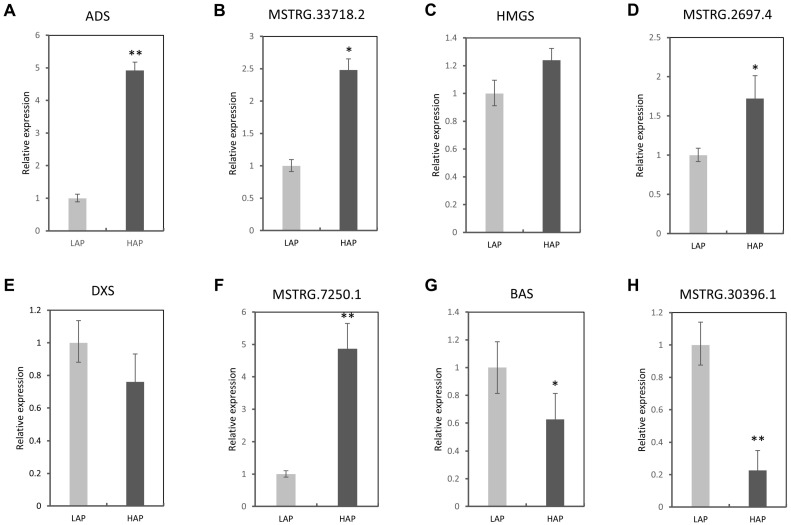
Validation of selected lncRNAs through quantitative RT-PCR in *Artemisia annua* HAP (high-artemisinin-producing type) and LAP (low-artemisinin-producing type) genotypes. (**A**–**H**) Comparative relative expression of both lncRNAs and corresponding target coding genes. *, ** refer to significant difference at *p* < 0.05 and 0.01, respectively.

From the differential expression of putative target genes, candidate genes related to artemisinin biosynthesis (*ADS*, *DXS*, *BAS* and *HMGS*) were selected for the quantitative RT-PCR experiment (Figure 6). The primer sequences are listed in Table S4.

Aside from *DXS* and its linked lncRNA MSTRG.7250.1, the three other genes and their corresponding lncRNAs showed similar trends, suggesting their effective interactions in the artemisinin biosynthesis pathway. Overall, the RT-PCR results were in line with the bioinformatics prediction.

## 4. Discussion

The present study is the first systematic screening of lncRNAs in *A. annua*, with a strategy based on a comparative analysis of two contrasting genotypes, HAP and LAP. The differential expression analysis in this study identified a distinct set of lncRNAs with predicted target genes that play roles in the biosynthesis of specialized plant metabolites, particularly sesquiterpenoid and triterpenoid compounds. This suggests a potentially crucial regulatory layer of lncRNAs in modulating the pathways responsible for these bioactive metabolites. The role of lncRNAs in metabolites biosynthesis has been previously uncovered in pharmaceutical plants including *Ganoderma lucidum* [32], *Cinnamomum camphora* [15], *Digitalis purpurea* [33] and *Salvia miltiorrhiza* [34].

In-depth lncRNAs and target gene correlation mining highlighted four genes involved in the artemisinin biosynthetic pathway, namely *ADS* (Amorpha-4,11-diene synthase), *DXS* (1-deoxy-D-xylulose-5-phosphate synthase), *BAS* (beta-amyrin synthase) and *HMGS* (3-hydroxyl-3-methyglutaryl CoA synthase)*. ADS* acts at an upstream step in the artemisinin biosynthetic pathway. It converts farnesyl diphosphate (*FPP*) into amorpha-4,11-diene, a key intermediate in the production of artemisinin (Figure 1A). The *ADS* gene is of particular interest in the field of synthetic biology and metabolic engineering for artemisinin production [35]. Any approach to the heterologous synthesis of artemisinin should start with the expression of *ADS*. The substrate promiscuity of *ADS* has also been exploited to develop chemoenzymatic strategies for artemisinin production [36,37]. In this study, the positive correlation between the expression of *ADS* and its associated lncRNA (MSTRG.33718.2) showed that *ADS* is a determinant for the high artemisinin accumulation in HAP.

*BAS* plays a key role in the biosynthesis of oleanane-type triterpene saponins by catalyzing the conversion of 2,3-oxidosqualene to β-amyrin [38]. This enzyme is part of a pathway that competes with artemisinin biosynthesis, making high *BAS* expression unfavorable for artemisinin production. In our study, we observed a strong down-regulation of *BAS* in HAP compared to LAP, in line with the expression pattern of the linked lncRNA MSTRG.30396.1. These findings suggest that suppressing competing pathways can enhance artemisinin accumulation in *A. annua*.

*DXS* is a key enzyme in the MEP (methylerythritol phosphate) pathway, which supplies precursors for artemisinin biosynthesis. It catalyzes the first step in the MEP pathway, which is the condensation of pyruvate and D-glyceraldehyde-3-phosphate to form 1-deoxy-D-xylulose-5-phosphate [3]. It is worth mentioning that DXS is considered a rate-limiting enzyme in the biosynthesis of terpenoids, including artemisinin [39]. Higher expression of *DXS* leads to increased artemisinin production, while suppression of *DXS* expression leads to decreased artemisinin production [39,40]. Therefore, *DXS* plays an important role in regulating the artemisinin biosynthetic pathway and is a potential target for manipulating this metabolic pathway. In this study, no significant differential expression of *DXS* was observed between HAP and LAP, although the linked lncRNA MSTRG.7250.1 was highly up-regulated in HAP. LncRNAs are known to regulate gene expression by interacting with mRNA stability, translation or degradation pathways [41]. In *Arabidopsis thaliana*, loss of function of the lncRNA *Npc48* induces leaf serrations and abnormal vegetative growth and it was found that it participates in the post-transcriptional regulation of *AGO1* [42]. We hypothesize that MSTRG.7250.1 could be affecting *DXS* activity through post-transcriptional regulation in *A. annua*.

*HMGS* is one of the key enzymes involved in the biosynthesis of artemisinin. It is part of the mevalonate (MVA) pathway, which provides the substrates isopentenyl pyrophosphate and dimethylallyl pyrophosphate for artemisinin biosynthesis [43]. *HMGS*, together with *DXR* (1-deoxy-D-xylulose-5-phosphate reductoisomerase), is a rate-limiting enzyme in the MVA pathway [44,45]. Inhibition of *HMGS* activity has been shown to reduce artemisinin production [3]. Therefore, HMGS plays an important role in the artemisinin biosynthetic pathway by providing the precursors for artemisinin biosynthesis. Here, we also found that the expression level of *HMGS* did not significantly change between HAP and LAP, although MSTRG.2697.4 was higher expressed in HAP. While lncRNA modulation of gene expression has been extensively studied, systematic investigations into post-transcriptional regulation by lncRNAs remain rare [46]. Further research on the post-transcriptional regulation of HMGS and DXS by their target lncRNAs in *A. annua* could provide new insights into the mechanisms of lncRNA-mediated regulation.

In the present study, quantitative PCR of candidate lncRNA-target gene pairs was performed to evaluate the bioinformatics results. The results showed a preferential high expression of the selected lncRNAs in the HAP genotype, indicating that the lncRNAs are more active in the high-producing artemisinin genotype compared to the low-producing genotype. Similar trends in relative expression were observed for both the lncRNA and their relative target genes, suggesting their high level of co-expression in the artemisinin biosynthesis process.

Functionally characterized lncRNAs play critical roles in plants, from regulating flowering to controlling lateral root formation [47,48]. lncRNAs exert their functions by interacting with DNA, RNA and protein molecules and modulating the expression levels of their targets [49]. They can stimulate or repress transcription through a variety of mechanisms, including epigenetic changes such as chromatin remodeling or histone modification [48]. In pharmaceutical plants, the role of lncRNAs in the biosynthesis of plant metabolites has become a hot topic of interest [12]. Overall, lncRNAs have emerged as important regulators of plant metabolite biosynthesis with potential applications in improving the production of bioactive compounds in medicinal plants. In the future, long-read sequencing technologies such as Nanopore or Pacbio have the potential to enhance the selection and analysis of lncRNA [50]. Overall, our study sheds light on the potential involvement of lncRNAs in artemisinin biosynthesis in *A. annua*, which could lead to a better understanding of the pathway and potentially new ways to improve artemisinin production. In-depth functional characterization of the selected lncRNAs is an area of investigation that could contribute to the increased production of artemisinin in *A. annua*.

## 5. Conclusions

RNA deep sequencing was conducted on *A. annua* genotypes with high and low artemisinin production, revealing four potential lncRNAs associated with key artemisinin biosynthetic genes (ADS, DXS, BAS and HMGS). This study provides valuable insights into lncRNAs that may influence artemisinin biosynthesis and sets the stage for future lncRNA research to support molecular-assisted improvement of artemisinin production in *A. annua*.

## Data Availability

The data presented in this study are available on request from the corresponding author(s).

## Supplementary Materials

The following supporting information can be downloaded at: https://www.mdpi.com/article/10.3390/life14111462/s1, Table S1: A summary of the raw sequencing data for the high-artemisinin-producing (HAP) and low-artemisinin-producing (LAP) *Artemisia annua* genotypes; Table S2: Differentially expressed lncRNAs cis-acting target genes in *Artemisia annua*; Table S3: Genes involved in artemisinin biosynthesis pathway; Table S4: List of markers used for PCR experiment; Figure S1: The chromatographs of sesquiterpenoids detected in HAP and LAP leaf extracts.
