# Supplementary figures and images for: Novel Differentially Expressed LncRNAs Regulate Artemisinin Biosynthesis in Artemisia annua
Source: Life (Basel). 2024 Nov 12;14(11):1462. doi: 10.3390/life14111462 (PMC11595271; doi:10.3390/life14111462)

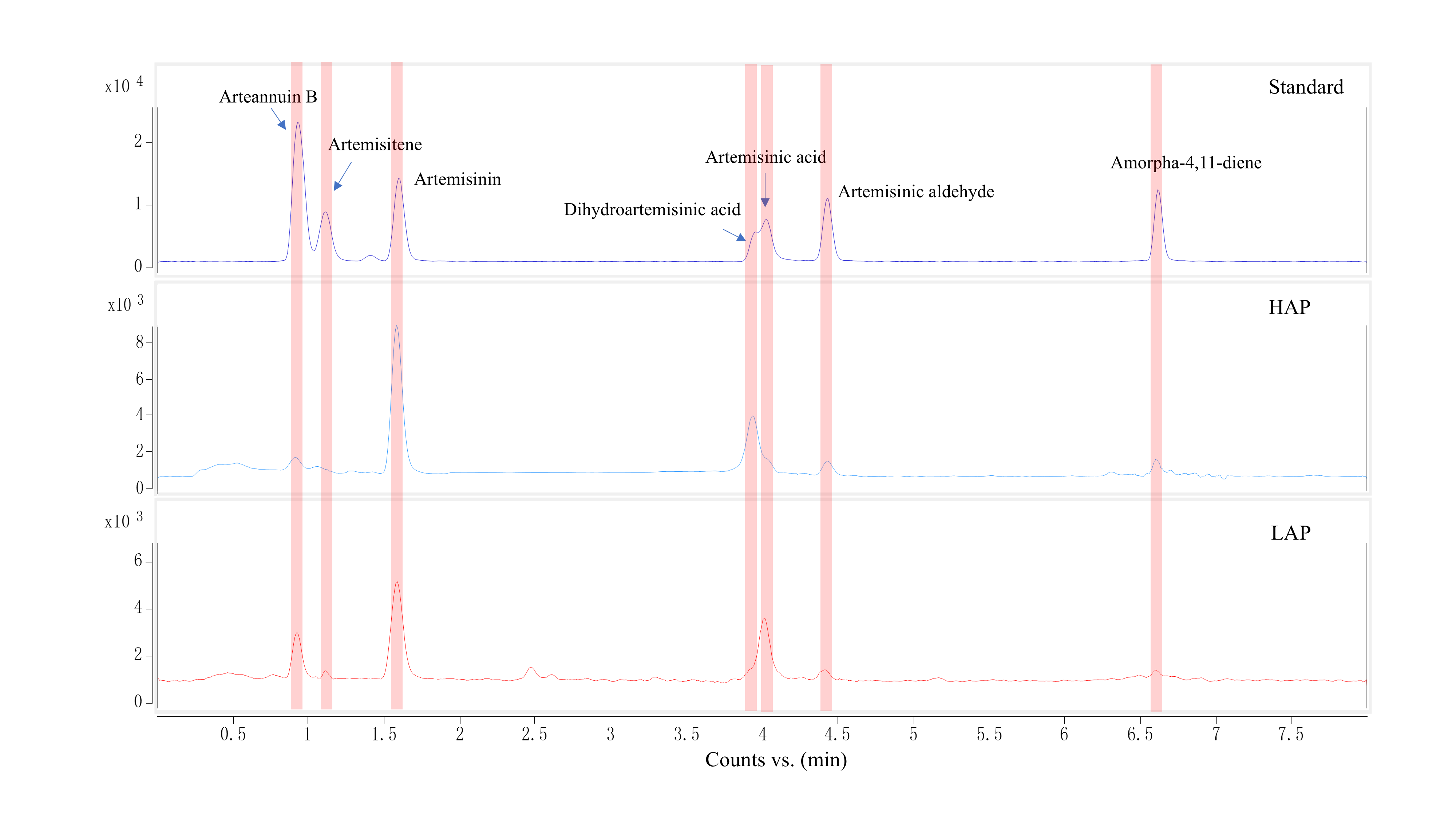

Supplement: Supplementary file 1 [file life-14-01462-s001.zip › Supplementary_Figure S1.tif]
